# Poorly Differentiated Neuroendocrine Tumor of the Esophagus with Hypertrophic Osteoarthropathy and Brain Metastasis: A Success Story

**DOI:** 10.7759/cureus.646

**Published:** 2016-06-19

**Authors:** Muhammad W Saif, Chandra Vethody

**Affiliations:** 1 Hematology/Oncology, Tufts Medical Center; 2 Medicine, Tufts Medical Center

**Keywords:** neuroendocrine tumor, esophagus, brain metastases, hypertrophic osteoarthropathy

## Abstract

Neuroendocrine carcinomas (NECs) of the esophagus are very rare. The majority of the patients with NECs present with metastasis. Paraneoplastic syndromes, such as syndrome of inappropriate secretion of anti-diuretic hormone and watery diarrhea-hypokalemia-achlorhydria syndrome, have been reported in previous reports. Esophageal NECs are related to a poor prognosis. A 38-year-old male with the histologic diagnosis of esophageal NEC, which initially manifested as hypertrophic osteoarthropathy (HOA), later developed brain metastases. He was initially treated with neoadjuvant chemotherapy consisting of cisplatin and etoposide followed by a partial esophagectomy in November 2009. At follow-up in February 2010, he complained of a headache that prompted imaging. MRI of the brain revealed a left frontal lobe lesion. Subsequently, he underwent a craniotomy and resection of the lesion. Pathological analysis revealed that the lesion was consistent with metastatic disease from the primary esophageal NEC. The patient underwent 40 Gy whole brain radiotherapy (WBRT), followed by two weeks of stereotactic radiation (SRS) to the tumor bed for an additional 12 Gy. During this time, his tumor marker neuron-specific enolase (NSE) initially dropped but later increased, which led us to offer him radiotherapy to the remaining esophagus to be followed by localized radiation to areas immediately adjacent to the surgical site, followed by six cycles of systemic chemotherapy consisting of cisplatin and irinotecan. Finally, his NSE normalized around the end of systemic chemotherapy. Surveillance imaging in 2015 - six years from initial diagnosis - showed no evidence of cancer. Of interest, treatment of the esophageal NEC also led to clinical resolution of his musculoskeletal symptoms, including his HOA. High-grade esophageal NECs are rare, aggressive, and have a poor prognosis. HOA can be a presenting sign associated with a high-grade esophageal NEC. The predominant site of metastatic spread is the liver, but metastases to rare sites, such as the brain, can occur, as was the case in our patient. Better survival has been reported for patients with locoregional disease compared to patients with distant metastases. However, multidisciplinary management can lead to an improved outcome in selected patients with distant metastases.

## Introduction

Neuroendocrine carcinomas (NECs) of the esophagus are very rare with a reported incidence of 0.05% - 2.4% of all esophageal cancers [1]. Pathological diagnosis can be challenging as it shares many clinicopathologic features with small cell esophageal carcinoma [2]. A pure NEC of the esophagus is extremely rare as the majority of the cases have mixed histology combined with squamous cell carcinoma, adenocarcinoma, or mixed glandular or squamous differentiation. Literature reports that esophageal NECs manifest as fungating or ulcerated masses, usually > 4 cm in diameter and involve the distal half of the esophagus [1-3]. The majority of the patients with NECs present with a metastasis [1-2]. Paraneoplastic syndromes, such as a syndrome of inappropriate secretion of antidiuretic hormone and a watery diarrhea-hypokalemia-achlorhydria syndrome, have been reported in previous reports [4].

Esophageal NECs are related to a poor prognosis [1-4]. No optimal treatment modality has been established due to the rarity of the disease. However, anecdotal data suggests a benefit of combined surgery, radiotherapy, and chemotherapy in these patients [1-3]. The World Health Organization (WHO) proposed new diagnostic criteria in 2010 for this disease and introduced grading.

We herein report a case of esophageal NEC, who initially manifested with hypertrophic osteoarthropathy (HOA) and subsequently developed brain metastases.

## Case presentation

Informed patient consent was obtained prior to treatment.

A 38-year-old male Caucasian postdoctoral researcher with a history of smoking as a teenager and hyperthyroidism presented to a rheumatologist with an eight-month history of progressive pain and swelling of multiple hand joints, knees, and ankles bilaterally. He drank alcohol only socially. However, he informed us that he might have been exposed to radiation while working in the laboratory. Laboratory workup showed no abnormalities. Initially, he was treated conservatively with nonsteroidal anti-inflammatory drugs (NSAIDs) and corticosteroids. However, his symptoms did not abate. Further evaluation by the rheumatologist, including bone scintigraphy with Tc-99m MDP (methylene diphosphonate), showed periosteal proliferation of the tibia and fibula consistent with hypertrophic osteoarthropathy (HOA). This diagnosis led to further investigations, and an attempt to look for an associated malignancy was initiated. He denied any systemic hormone syndromes, such as flushing or diarrhea. CT scans of the abdomen, pelvis, and chest suggested an esophageal tumor. Positron emission tomography-computed tomography (PET/CT) was performed and revealed an ^18^F-fluorodeoxyglucose (^18^F-FDG) avid lesion in the distal esophagus. A subsequent esophagogastroduodenoscopy (EGD) was performed and biopsies of the mass were obtained. The histologic analysis of the biopsies showed esophageal NEC with round to spindle-shaped cells with scanty cytoplasm, granular nuclei, and inconspicuous nucleoli. Immunohistochemical (IHC) analysis revealed a population of cells positive for synaptophysin, CD56, chromogranin, K11, and epithelial membrane antigen (EMA). The sample was negative for TTF-1, CK5, Lu5, and CK20. A proliferative index (Ki-67) was 80%. Therefore, the final diagnosis was consistent with a poorly differentiated (high-grade) NEC of the esophagus and staged as a T2 N1 M0 esophageal cancer, according to the TNM/staging classification for esophageal carcinomas. Peripheral blood testing showed normal levels of complete blood counts, chemistry, and all the basic biochemical parameters, including the tumor markers (CEA 1.6 ng/mL, CA19-9 12 U/mL, serotonin, and chromogranin), except for a neuron-specific enolase (NSE) of 15.5 ng/mL (normal: < 8.6 mcg/mL).

After discussion in a multidisciplinary meeting, the patient was started on neoadjuvant chemotherapy consisting of cisplatin and etoposide. He completed three cycles of therapy (three months) prior to proceeding with a partial esophagectomy with a gastric pull-through in November 2009. He recovered well in the postoperative setting. At his three month follow-up in February 2010, he complained of a headache that prompted imaging. MRI scan of the brain revealed a left frontal lobe lesion. He subsequently underwent a craniotomy and resection of the lesion. Pathological analysis revealed that the lesion was consistent with metastatic disease from the primary esophageal NEC. Further assessment with somatostatin scintigraphy did not reveal sites of uptake. The patient underwent whole brain radiotherapy (WBRT) with 40 Gy followed by two weeks of stereotactic radiation (SRS) to the tumor bed for an additional 12 Gy. During this time, his NSE rose to 37.6 ng/mL on May 21, 2010, dropped to 13 ng/mL, and then again rose to 41 ng/mL.

The patient received a total of 54 Gy external beam radiation to the remaining esophagus, including the adjacent surgical site, from mid-April until the end of May 2011. In addition, an additional boost of 45 Gy was administered to the region of the chest. This was followed by six cycles of systemic chemotherapy consisting of cisplatinum and irinotecan. Finally, his NSE normalized at 5.6 ng/mL around the end of the systemic chemotherapy. After that, he was closely monitored with scans every three months. Over the course of the past five years, his clinical status has remained largely stable. Periodic laboratory surveillance measuring the NSE has remained below the upper limit of normal. Chromogranin A levels have fluctuated between normal and mildly elevated levels. Surveillance imaging has been negative for recurrence of disease within the brain and thorax when last seen in 2015, six years from his initial diagnosis. Of interest, treatment of esophageal NEC also led to clinical resolution of his musculoskeletal symptoms, including HOA.

## Discussion

Esophageal NECs are of exceedingly rare occurrence [1-2]. Most patients present with distant metastases and, hence, carry a poor outcome. No formal staging classification for esophageal NEC exists, but physicians use TNM/staging classification for esophageal carcinomas in clinical practice for the purpose of aiding in the treatment decision. Histological grade also dictates prognosis in addition to staging. Due to the rarity of this entity of esophageal cancer, there are no established treatment protocols, and data is mostly derived from case reports and small case series.

As is for other types of tumors, esophagectomy with curative intent is commonly used in locoregional disease. Unfortunately, surgery alone is not commonly associated with long-term survival. Similarly, radiotherapy alone also led to dismal outcome [5]. Therefore, investigators introduced adjuvant chemoradiotherapy as well as neoadjuvant chemoradiotherapy – both have also been reported in the review of the literature with improved outcome in these patients. Platinum agents, such as cisplatin, make the backbone of these chemotherapy regimens. Other agents employed in these patients included etoposide, irinotecan, cyclophosphamide, amrubicin, and doxorubicin [6-8]. Extrapolated from small lung cancer data, combination doublets of cisplatin/etoposide and irinotecan/etoposide are the most commonly used regimens as was also used in our patient. Although octreotide and lanreotide both have shown activity against NECs of the gastrointestinal tract, no specific data exists at present for esophageal NEC. Therefore, a combination modality treatment under a multidisciplinary team should be involved in the care of these patients.

Interestingly, our patient first manifested HOA. HOA is a syndrome characterized by proliferative changes in the skin and skeleton. In addition, it may also comprise of proliferative periostitis of the long bones, oligopoly synovitis, and digital clubbing. There are two types of hypertrophic osteoarthropathy: primary and secondary. Only 3-5% of patients have primary HOA while the remaining 95-97% have secondary HOA, commonly associated with many disease conditions of the cardiovascular system and hepatobiliary gastrointestinal system as well as in malignancies. Lung and breast cancers are the most common tumors that produce HOA. HOA is extremely rare in patients with esophageal cancer; however, medical literature indicates that HOA may be the first manifestation of esophageal cancer in a few cases and may delay the diagnosis of fatal cancer if not diagnosed early [9]. Our case further underlines the fact that an uncommon presentation, such as HOA, may be the first symptom of an underlying malignancy, such as in our case, and early recognition can lead to a timely diagnosis. Bone scintigraphy with Tc-99m MDP [methylene diphosphonate] is a sensitive study in the detection of HOA.

Another interesting finding in our patient was the elevated NSE, which was utilized as a tumor marker. NSE is present in the cytoplasm of most neuroendocrine cells [10]. The sensitivity and specificity of NSE in carcinoid tumors are 33% and 100%, respectively. NSE is also useful for the follow-up and monitoring of patients with neuroendocrine tumors (NET) as previously suggested.

## Conclusions

In summary, high-grade esophageal NECs are rare, aggressive, and have a poor prognosis. HOA can be a presenting sign associated with a high-grade esophageal NEC. The predominant site of metastatic spread is the liver but rare metastatic spread to sites, such as the brain, can occur, as seen in our patient. Chromogranin A levels are the commonly used tumor markers, but NSE can be used in selected cases. The prognosis of esophageal NEC correlates with the grade and stage of the disease. Better survival has been reported for patients with locoregional disease compared to patients with distant metastases. However, multidisciplinary management can lead to an improved outcome in selected patients with distant metastases. Combined treatment consisting of chemotherapy, radiotherapy, and surgery can offer an effective outcome. Platinum-based systemic chemotherapy forms the backbone of treatment for these NET.
